# Towards a clinical decision protocol for therapeutic plasma exchange based on biomarker patterns and machine learning

**DOI:** 10.1186/s12911-026-03484-3

**Published:** 2026-04-16

**Authors:** Nicoleta Sgăvârdea, Darian Onchiş, Fabian Galiş, Ciprian Gindac, Mirela Poroşnicu, Adelina Marinescu, Codruţa Istin, Voichiţa Lăzureanu

**Affiliations:** 1 “Victor Babes” University of Medicine and Pharmacy, Eftimie Murgu Square 2, Timisoara, Timis 300041 Romania; 2 Faculty of Computer Science, West University of Timisoara, Vasile Pârvan Blvd. 4, Timisoara, Timis 300223 Romania; 3 Faculty of Automation and Computing, Politehnica University of Timisoara, Victory Square 2, Timisoara, Timis 300006 Romania; 4 Department of Anaesthesia and Intensive Care, “Victor Babes” University of Medicine and Pharmacy, Eftimie Murgu Square 2, Timisoara, Timis 300041 Romania; 5 Department of Infectious Diseases, “Victor Babes” University of Medicine and Pharmacy, Eftimie Murgu Square 2, Timisoara, Timis 300041 Romania

**Keywords:** Therapeutic plasma exchange, COVID, Machine learning, Decision tree, Apriori, Interpretability

## Abstract

**Introduction:**

Therapeutic plasma exchange (TPE) is increasingly used as an adjunctive intervention in severe, hyperinflammatory critical illness, including COVID-19, yet clinical guidance remains syndromic and evidence is heterogeneous. We present an integrated, interpretable machine-learning framework designed to support protocolizable TPE decision-making by identifying biochemical phenotypes associated with short-horizon laboratory response to TPE. Our dataset consists of real-world intensive care unit cases and captures the treatment heterogeneity and operational constraints that a workable institutional protocol must accommodate, being well-suited as a “protocol seed” for iterative validation.

**Methods:**

We jointly analyze a COVID-19 cohort and a non-COVID comparator cohort receiving TPE. Three decision trees were constructed to represent: (1) global biochemical improvement, (2) strict improvement dependent on key inflammatory/coagulation markers, and (3) early interleukin-6 (IL-6) response. The models revealed distinct favorable phenotypes—particularly patients with IL-6 > 86 pg/mL, lactate dehydrogenase (LDH) >346 U/L, lymphopenia, and fibrinogen ≤ 8.3 g/L. Apriori analysis further identified high-confidence patterns linking high values of IL-6 and LDH with TPE responsiveness. We constructed a unified four-tier clinical algorithm for candidate stratification.

**Results:**

The resulting logic aligns with how TPE has been applied in practice in published severe COVID-19 series and trials, where candidate selection typically targets cytokine release syndrome-like phenotypes, organ dysfunction, and hyperinflammatory biomarker profiles. We further contextualize these findings against American Society for Apheresis guidance and COVID-era operational considerations, demonstrating convergence while adding quantitative biomarker thresholds.

**Conclusion:**

These findings support two complementary perspectives of benefit: (i) an algorithmic framework that provides transparent, protocol-ready guidelines, and (ii) a phenotype-based clinical approach consistent with observed post-TPE marker changes in severe COVID-19 cases. Larger multicenter validation is warranted; however, the present work provides a practical foundation for protocol construction and auditable decision support in settings already performing TPE.

## Introduction

Therapeutic plasma exchange (TPE) is a resource-intensive extracorporeal intervention that removes circulating plasma components and replaces them with albumin and/or plasma. It is used in multiple immune-mediated and inflammatory conditions, where it can reduce pathogenic antibodies, immune complexes, acute-phase reactants, and other mediators of systemic inflammation  [1, 2]. During the COVID-19 pandemic, TPE gained attention as an adjunctive strategy for selected critically ill patients with hyperinflammatory or thrombo-inflammatory features, motivated by early descriptions of cytokine-driven deterioration and endotheliopathy  [3–7].

However, despite plausible mechanisms and multiple reports describing improvements in inflammatory markers and oxygenation after TPE in severe COVID-19, the clinical evidence base remains mixed and context-dependent, ranging from small series to early-terminated trials  [8–11]. In parallel, professional guidance is necessarily conservative. The American Society for Apheresis (ASFA) guidelines are structured around indication categories and grades of recommendation, and COVID-era ASFA operational considerations emphasize prioritization and selective use under constrained resources, while explicitly noting potential risks such as unintended removal of immunoglobulins and concurrently administered therapies  [1, 12, 13]. In practice, clinicians are therefore left with a high-stakes selection problem: identifying which patients are most likely to derive meaningful benefit from TPE, and how to monitor response in a timely, auditable manner.

This work addresses that gap using a deliberately interpretable analytic approach and a clinically grounded endpoint. Although our database is small, it is composed of real intensive care unit (ICU) cases treated with TPE and therefore reflects the operational realities—heterogeneous disease severity, evolving practice patterns, and incomplete information—that any viable local protocol must handle. Rather than claiming definitive efficacy, we treat this dataset as a protocol seed: sufficient to derive an initial, explicit decision logic that can be prospectively tested, audited, and refined.

We emphasize two major perspectives:

1. **An algorithmic approach** (protocolizable guidance). We derive transparent rules that specify candidate-selection criteria and near-term monitoring signals for biochemical response. The goal is not a black-box predictor, but a set of human-auditable decision pathways that can be translated into protocol elements (triage thresholds, escalation/de-escalation logic, monitoring cadence).
2. **A medical perspective** (phenotype clarification). By highlighting biomarker configurations that segregate responders from non-responders, the framework contributes to clinical interpretation of which inflammatory phenotypes appear most consistent with post-TPE biochemical shifts reported in severe COVID-19 experience  [9, 10, 14, 15].

**Fig. 1:**
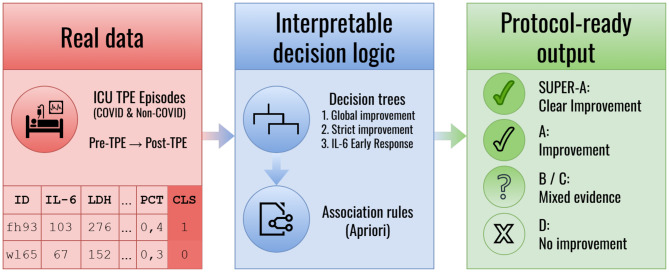
Process overview: from real TPE episodes to protocol-ready conclusions

Interpretable modeling is essential when decision support is intended to be operationalized as a clinical protocol. This emphasis is consistent with the broader interpretability literature that seeks to make model structure directly understandable, even in settings where conventional supervision is limited or absent  [16]. Accordingly, we propose an integrated decision-tree and association-rule framework that combines predictive structure with interpretability, and we contextualize the resulting decision logic against ASFA guidance and COVID-era ASFA considerations to support protocol development for TPE in severe inflammatory critical illness. An overview of our process is shown in Fig. 1. Abbreviations used throughout the manuscript are summarized in Table 1.

**Table 1:** Abbreviations used in this manuscript

Abbreviation	Definition
ARDS	Acute respiratory distress syndrome
ASFA	American Society for Apheresis
AUC	Area under the curve
CI	Confidence Interval
COVID-19	Coronavirus disease 2019
CRP	C-reactive protein
CRS	Cytokine release syndrome
ICU	Intensive care unit
IL-6	Interleukin-6
LDH	Lactate dehydrogenase
PCT	Procalcitonin
ROC	Receiver operating characteristic
SARS-CoV-2	Severe acute respiratory syndrome coronavirus 2
TPE	Therapeutic plasma exchange

## Literature review

Severe COVID-19 frequently involves dysregulated inflammation and downstream organ dysfunction, with inflammatory and endothelial injury markers associated with clinical deterioration  [3–5, 7, 17]. This biology motivated the exploration of TPE as an adjunctive intervention in selected critically ill patients, aiming to attenuate circulating inflammatory mediators and modulate coagulation- and viscosity-related abnormalities.

A summary of TPE studies can be observed in Table 2. Across COVID-19 TPE reports, candidate selection has most commonly targeted Chronic Rhinosinusitis (CRS) phenotypes, severe hypoxemia/Acute Respiratory Distress Syndrome (ARDS), multi-organ dysfunction, and marked inflammatory marker elevation. In a pilot series of severe COVID-19 patients with ARDS and shock features, multi-session TPE was associated with reductions in interleukin-6 (IL-6), C-reactive protein (CRP), ferritin, and lactate dehydrogenase (LDH) alongside improvements in the sequential organ failure assessment (SOFA) score and oxygenation indices  [9]. In a CRS-focused series using repeated exchanges over approximately one week, improvements in oxygenation parameters and broad cytokine reductions were described  [14]. A propensity-matched retrospective analysis also reported improved outcomes with adjunctive TPE in COVID-19-triggered CRS and suggested timing sensitivity (earlier initiation performing better)  [11]. More recently, an early-terminated randomized trial reported shorter ICU length of stay and fewer mechanical ventilation days in the TPE arm, with biomarker improvements including IL-6- and coagulation-related markers  [10]. Mechanistic or phenotype-specific reports further underscore that the “TPE target” may differ across subgroups. A hyperviscosity case series described rapid viscosity correction and reductions in fibrinogen and inflammatory markers after TPE  [18]. Separately, plasma exchange was used as rescue therapy in patients with neutralizing autoantibodies against type I interferons, demonstrating reduction of those autoantibodies while preserving anti–SARS-CoV-2 antibody levels in the reported cases  [19].

**Table 2:** Summary of TPE studies

Study	Target phenotype	TPE regimen	Reported effects	Key limitation
Faqihi 2020 [9]	Life-threatening COVID-19; ARDS/shock	Multi-session; 1.0–1.5 PV; 5–7 sessions	$\downarrow$IL-6/CRP/ferritin/LDH; $\uparrow$P/F; $\downarrow$SOFA	Small; nonrandomized
Gluck 2020 [14]	CRS (Penn 3–4)	5 exchanges over ~8 days	Improved oxygenation; $\downarrow$CRP and cytokines	Small; nonrandomized
Kamran 2021 [11]	COVID-19 CRS	Not consistent	Improved survival/CRS resolution; timing sensitivity	Residual confounding
Faqihi 2021 [10]	Life-threatening ICU COVID-19	Protocolized	Shorter ICU LOS and MV days; biomarker improvements	Limited power
Truong 2021 [18]	Hyperviscosity	Daily; 2–3 TPEs	Rapid viscosity correction; $\downarrow$fibrinogen/D-dimer/CRP	Very small
de Prost 2021 [19]	Anti–type I IFN autoAbs	Several sessions	$\downarrow$anti-IFN autoAbs; preserved anti–SARS-CoV-2 Abs	Selected subgroup

Collectively, these studies emphasize that TPE in COVID-19 has been deployed primarily as an adjunct for severe phenotypes characterized by hyperinflammation, organ dysfunction, or distinct immunologic abnormalities—rather than as a general therapy for mild disease. Professional guidance reflects this uncertainty and prioritizes selective use. ASFA guidelines classify indications using evidence-informed categories and grades  [1]. COVID-era ASFA operational considerations highlight resource stewardship and reiterate that procedures with stronger evidence/priority should proceed preferentially, while emphasizing cautions relevant to TPE, including the possibility of removing concurrently administered therapies and immunoglobulins, and the need to avoid indiscriminate use (particularly in mild presentations)  [12, 13]. Notably, preliminary COVID-specific discussions in the transfusion/apheresis literature summarize TPE for severe COVID-19 as an ASFA Category III-style context (role not established; decision individualized) and recommend using severity scores and inflammatory markers for selection and monitoring  [15].

Given the combination of mechanistic plausibility, heterogeneous and evolving evidence, and conservative guideline framing, an interpretable decision-support framework that identifies response-associated phenotypes using real-world TPE-treated data can provide immediate value: it can make implicit selection heuristics explicit, auditable, and translatable into a protocol that can be prospectively tested.

## Methods

Two retrospective cohorts were analyzed: (1) patients with confirmed COVID-19 infection and (2) non-COVID patients, all of whom underwent TPE. Both cohorts included extensive biochemical profiling before and after TPE. Key inflammatory markers included IL-6, CRP, LDH, procalcitonin (PCT), fibrinogen, ferritin, lymphocyte counts, and other laboratory parameters. For machine-learning analysis, each marker was paired as pre-procedure and post-procedure values. Clinical data were collected at the Victor Babes Infectious Diseases Hospital, Timisara, Romania. The recordings for our comparative study for patients with COVID pathology span the period March 2020 to December 2021 and those with non-COVID pathology the period 2014 until 2024, with a sample size of *N* = 60. The youngest patient with COVID pathology who underwent the TPE procedure was 22 years old, and the oldest was 80 years old, with a mean age of 51 years. From our database, it can be observed that this procedure was more frequently required among male patients. In the non-COVID patient database, the youngest patient who required the TPE procedure was 20 years old, and the oldest was 80 years old, with a mean age of 50 years.

Clinical and laboratory variables were collected at two predefined time points. Baseline measurements (B for before) correspond to values recorded immediately before the initiation of therapeutic plasma exchange (TPE), while follow-up measurements (A for after) correspond to values obtained after the TPE procedure or within the first 24 hours following the intervention. Variables marked with the suffix “0.1” in the dataset represent the A measurements. The list with core predictors of demographic variables, vital signs, and laboratory biomarkers collected at both time points is presented in Table 3, while inflammatory biomarkers and clinical severity scores used for patient assessment are summarized in Table 4. Patient outcomes, comorbidities, and therapeutic interventions are reported in Table 5. This structure allows the evaluation of biomarker dynamics and physiological response to plasma exchange, which were subsequently used as input features for the machine learning models.

**Table 3:** Demographic characteristics and key physiological parameters measured before (B) and after (A) therapeutic plasma exchange

Patient Characteristics						Clinical Parameters					
Name	Sex	Age	Weight	Height	TPE Day	Temperature		PaO_2_/FiO_2_		Respiratory Rate	
						B	A	B	A	B	A

**Table 4:** Selected laboratory biomarkers measured at baseline (B) and after therapeutic plasma exchange (A)

Core Laboratory Biomarkers							
pH		Lactate		Leukocytes		Fibrinogen	
B	A	B	A	B	A	B	A

**Table 5:** Inflammatory biomarkers, severity scores and clinical outcomes used for model development and evaluation

Inflammatory Markers, Severity Scores and Outcomes								
CRP		Ferritin		APACHE II		SOFA		Survival
B	A	B	A	B	A	B	A	

Regarding the background of the data we used in our study, the COVID-19 pandemic accelerated the negative demographic trends in Romania ie. population decline, negative natural growth, and population aging, with a strong impact in 2020–2021. In Timis County, the population loss during the immediate pandemic period was followed by stabilization and modest growth in 2022–2023, suggesting a somewhat more resilient local demographic dynamic.

Timişoara has a population of approximately 250,000–300,000 inhabitants, with a predominantly adult demographic structure. The average age is around 40 years, indicating a relatively balanced distribution between young, adult, and elderly populations, with a slight aging trend similar to the demographic evolution at the national level.

### Outcome definitions

Three separate outcome variables (“improvement labels”) were created to capture different definitions of biochemical response:

1. T1 – Global Improvement: Reduction in all or majority of inflammatory markers.
2. T2 – Strict Improvement: Required reductions in CRP and LDH, with emphasis on PCT and fibrinogen.
3. T3 – Early IL-6 Response: Binary indicator of IL-6 reduction after TPE.

These definitions allow modelling of broad, strict, and early-response phenotypes.

For T1, the inflammatory marker set was defined by paired pre/post availability in the dataset (fibrinogen, D-dimer, LDH, ferritin, procalcitonin, IL-6, and CRP). Let *m* be the number of these markers with both pre- and post-procedure values available for a given episode, and let *d* be the number that decreased (post < pre). We define “majority” as $d \ge \lfloor m/2 \rfloor + 1$. The T1 label is positive if either *d* = *m* (all available markers decreased) or *d* meets the majority criterion; if *m* = 0, the label is left undefined.

Missing values were handled per endpoint definition. For T1, only markers with both pre and post values contributed to the majority criterion, and the T1 label was left undefined if no paired inflammatory markers were available. For T2, strict improvement was left undefined if either CRP or LDH lacked paired pre/post values. For T3, early IL-6 response was left undefined if IL-6 lacked paired pre/post values.

### Decision-tree modelling

For each label (T1, T2, T3), a separate decision tree was trained using pre-procedure features only. The models were deliberately restricted (with a maximum depth of 3–4) to ensure interpretability consistent with clinical protocol needs.

1. **T1 - Global Improvement Tree** (Fig. 2) with predictors: LDH, Absolute Lymphocyte Count. Its rules can be summarised as:
  LDH > 346 U/L $$\rightarrow$$ improvement;
  LDH ≤ 346 U/L, lymphocytes $$0.53 \times 10^{9}\!/L$$$$\rightarrow$$ improvement (no improvement for lower values).

**Fig. 2:**
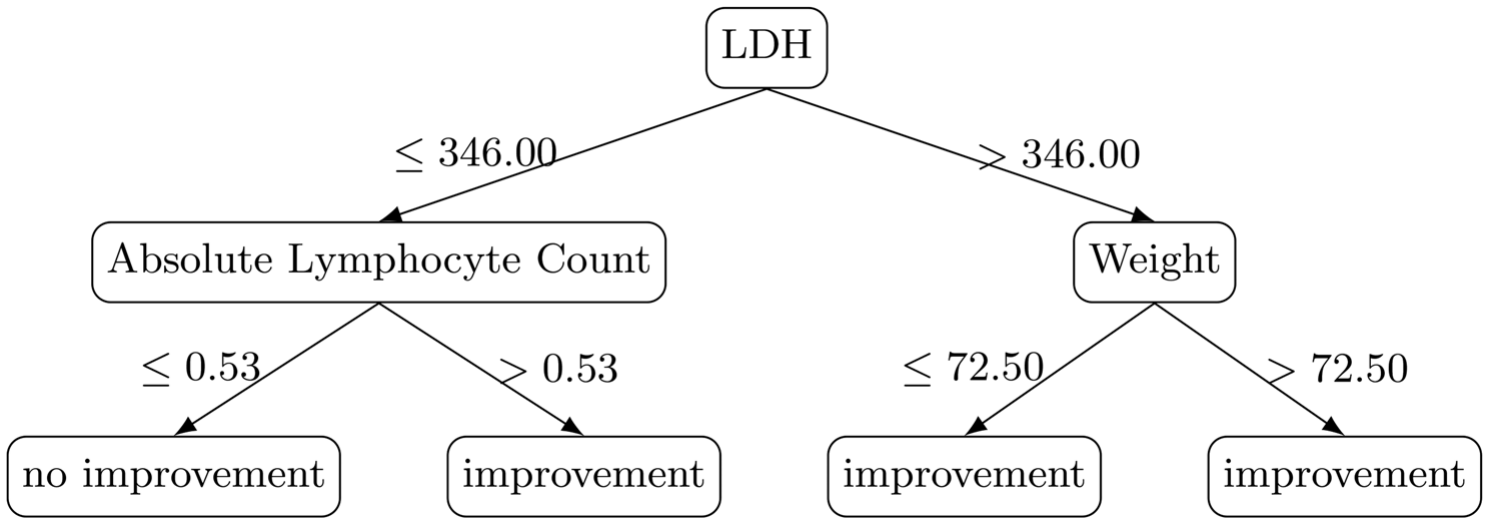
Extracted decision rules of tree 1
2. **T2 - Strict Improvement Tree** (Fig. 3) with predictors: PCT, CRP, LDH. Its rules can be summarised as:
  PCT ≤ 0.16 ng/mL $\rightarrow$ lack of strict improvement.
  PCT > 2.85 ng/mL and/or CRP > 81.26 mg/L $\rightarrow$ strong improvement.

**Fig. 3:**
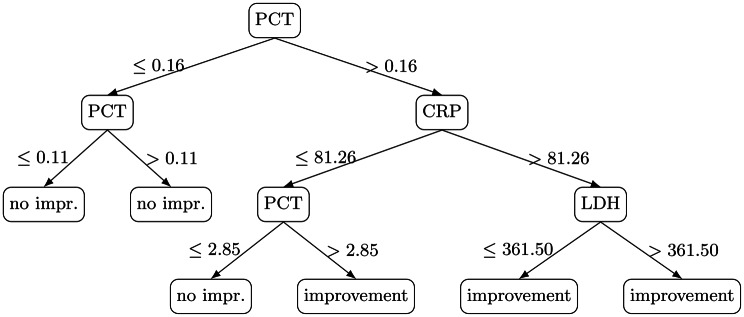
Extracted decision rules of tree 2
3. **T3 - IL-6 Early Response Tree** (Fig. 4) with predictors: IL-6, Fibrinogen. Its rules can be summarised as:
  IL-6 ≤ 7.24 pg/mL $\rightarrow$ early improvement;
  IL-6 $\in$ (7.24 pg/mL, 86.15 pg/mL] $\rightarrow$ no early improvement;
  IL-6 > 86.15 pg/mL + fibrinogen ≤ 8.31 g/L $\rightarrow$ early improvement;
  IL-6 > 86.15 pg/mL + fibrinogen > 8.31 g/L $\rightarrow$ no improvement.

**Fig. 4:**
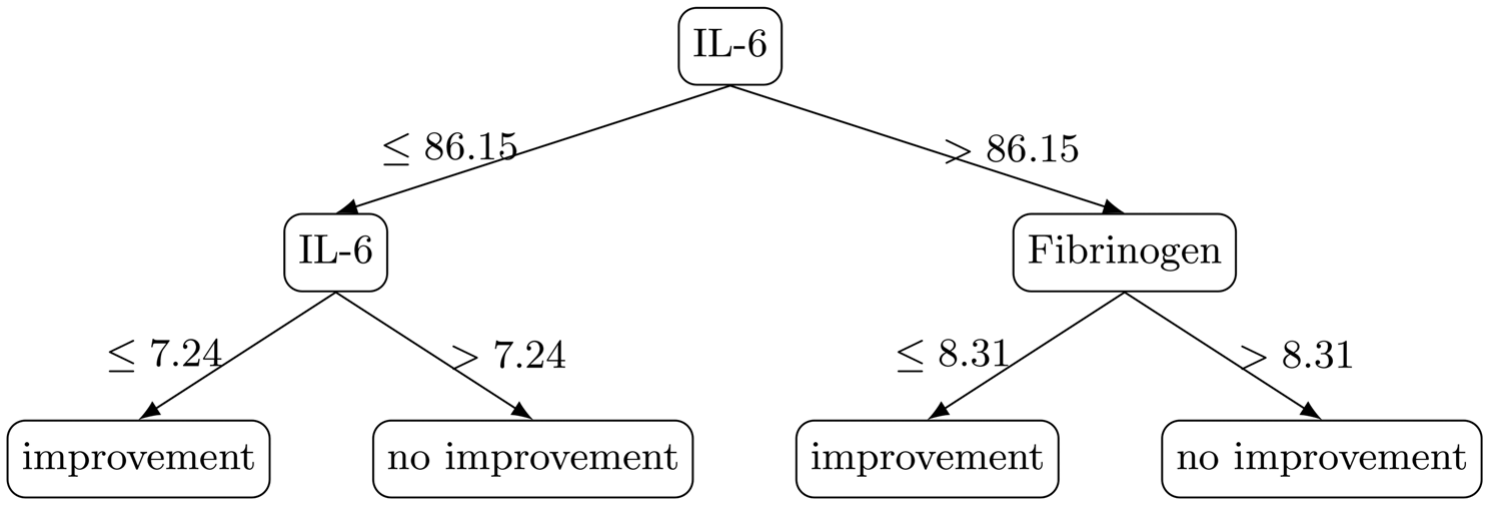
Extracted decision rules of tree 3

Each label represents a different operational definition of short-horizon biochemical response: T1 captures broad, multi-marker improvement; T2 enforces a stricter definition anchored on CRP and LDH with additional emphasis on PCT/fibrinogen; and T3 isolates early IL-6 response as a rapid, clinically salient signal. For each tree, pre-procedure values were provided as candidate predictors. Candidate feature sets differed by label: T1 was trained on the available pre-procedure physiologic and laboratory features in the dataset, whereas T2 and T3 were trained on a smaller, marker-focused candidate set centered on the markers implicated in their respective endpoint definitions. Because trees were deliberately constrained to shallow depth for protocol interpretability, the final extracted trees use a small subset of the candidate predictors.

The classification performance metrics for each decision tree, along their confusion matrix, can be observed in Tables 6, 7 and 8.

**Table 6:** Tree 1 confusion matrix accuracy: 0.714 (95% CI: 0.29–0.96) F1 score: 0.833 (positive class)

	Pred.		
True	0	1	Total
0	0	1	1
1	1	5	6
Total	1	6	7

**Table 7:** Tree 2 confusion matrix accuracy: 0.625 (95% CI: 0.24–0.91) F1 score: 0.667 (positive class)

	Pred.		
True	0	1	Total
0	2	3	5
1	0	3	3
Total	2	6	8

**Table 8:** Tree 3 confusion matrix accuracy: 0.625 (95% CI: 0.03–0.65) F1 score: 0.667 (positive class)

	Pred.		
True	0	1	Total
0	2	3	5
1	0	3	3
Total	2	6	8

Performance metrics are reported for transparency and must be interpreted cautiously given the small sample size and class imbalance in several endpoints. Although we restricted model complexity (shallow trees with minimum leaf sizes) to reduce overfitting, estimates from a single train/test split can remain optimistic in this regime. We therefore report exact binomial 95% confidence intervals (CI) for test-set accuracy alongside point estimates.

### Augmenting decision tree predictions with association rule mining

Apriori is a widely used association-rule mining algorithm designed to identify frequent itemsets and derive implication rules of the form “if A, then B”. Its key principle states that if a given itemset is infrequent, all of its supersets must also be infrequent, enabling efficient pruning of the search space  [20].

In our context, association-rule mining is not used as a standalone classifier but as a complementary post-model refinement layer. Decision trees estimate individualized probabilities of biochemical response based on global optimization criteria. However, tree-based models may underweight rare but clinically meaningful phenotypes due to sample-size constraints and impurity minimization.

Apriori association rules, in contrast, identify frequent, high-confidence co-occurring biomarker patterns without enforcing a hierarchical decision structure. In this study, Apriori consistently identified a severe inflammatory phenotype—characterized by very high IL-6, elevated LDH, and lymphopenia—that demonstrated near-uniform biochemical response to therapeutic plasma exchange.

#### Unified algorithm construction

**Fig. 5:**
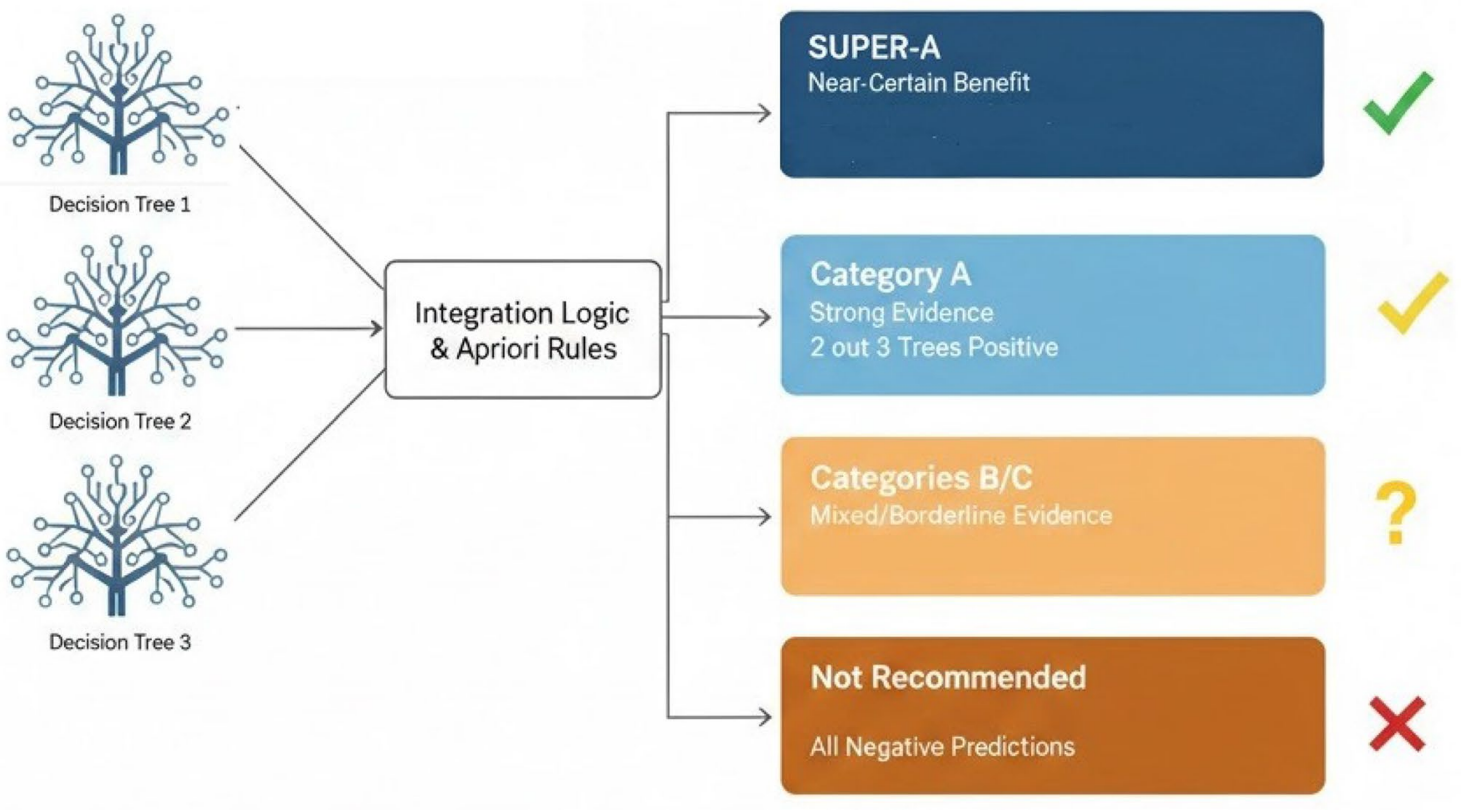
Unified TPE algorithm for predicting the likelihood of laboratory response

The outputs of the three decision-tree models were integrated into a unified four-tier clinical decision framework as shown in Fig. 5. The highest recommendation level (SUPER-A) was assigned to patients demonstrating concordant positivity across all three models or satisfying high-confidence association rules derived from Apriori analysis, indicating a near-certain biochemical response. Category A represented strong evidence for benefit, defined by concordant positive predictions from two of the three models. Categories B and C reflected mixed or borderline evidence, characterized by partial or inconsistent model agreement. Category D corresponded to uniformly negative predictions across all models, for which therapeutic plasma exchange was not recommended.

The Apriori algorithm was applied to a discretized dataset using bin thresholds derived from the decision trees, in order to also establish non-linear connections between the biomarkers. Rules with support ≥ 0.2, confidence ≥ 0.3, and lift ≥ 1.2 were extracted. Given that the Apriori-augmented analysis is conducted on the *N* = 28 episodes used in the T1 model table construction, the stated minimum support threshold ($\geq 0.2$) implies that each reported rule is supported by at least 6 episodes in this cohort. We emphasize that these patterns represent high-certainty co-occurrence signals within this retrospective dataset and should not be interpreted as universal responder phenotypes without external validation.

Key high-confidence rules:

- (IL6_very_high & LDH_high) $\rightarrow$ (T3 = 1) with confidence 0.93
- (T1 & IL6_very_high & Lymph_low) $\rightarrow$ (T3 = 1) with confidence 1.00
- (IL6_very_high & LDH_high) $$\rightarrow$$ (T1 = 1 & T3 = 1)

Rules were incorporated to identify “super-responders” (SUPER-A class).

**Fig. 6:**
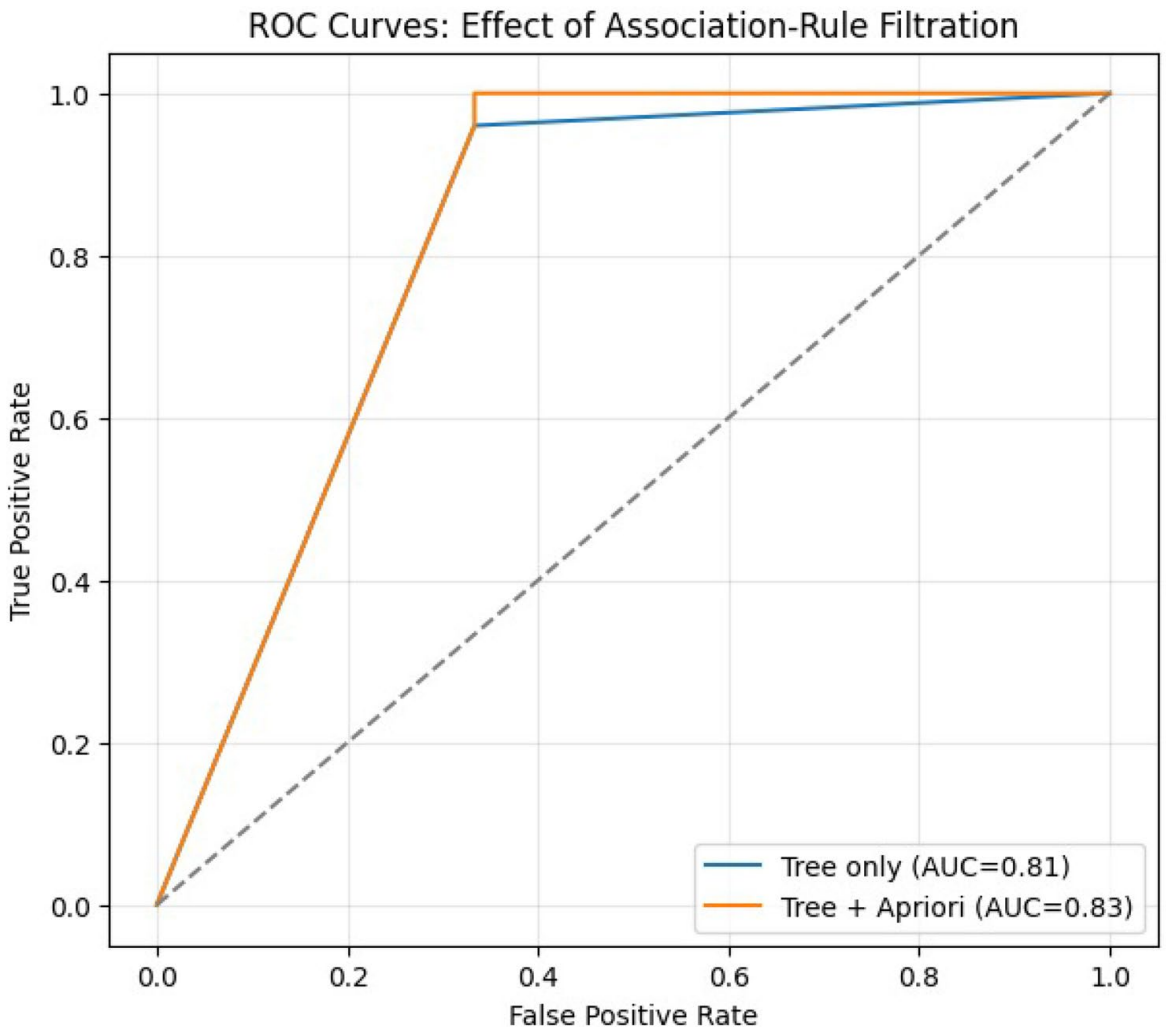
Receiver operating characteristic (ROC) curves computed from model-predicted probabilities across all thresholds, comparing the baseline decision-tree probabilities with the probabilities adjusted by the association rules. The AUC changes from 0.813 to 0.827 (values best interpreted as cohort-internal discrimination estimates rather than definitive generalization performance). At a fixed threshold of 0.5 the confusion matrix remains unchanged

By integrating these rules as a probability-adjustment filter, the combined model selectively increased predicted response probabilities for patients belonging to biologically coherent, high-confidence responder clusters. In this cohort, association-rule filtration produces a change in the ROC–AUC (Fig. 6), while thresholded classification metrics at 0.5 and net reclassification remain unchanged; accordingly, we treat this layer as an interpretable, phenotype-focused refinement signal rather than performance gain.

Conceptually, the decision trees captured global decision boundaries, while the association rules captured local phenotypic certainty regions. Their integration therefore enhanced model calibration and clinical relevance, particularly for identifying super-responder subgroups that are underrepresented in guideline-based or purely supervised models.

## Results and clinical findings

All three decision trees revealed highly interpretable branching logic aligned with known inflammatory pathophysiology. All numeric thresholds reported below are data-derived split points learned by shallow decision trees trained on this retrospective cohort, presented to make the protocol-seeding logic explicit and auditable.

1. **T1 – Global Improvement**: The identification of LDH as the primary split aligns with LDH’s role as a marker of tissue injury. Patients with LDH > 346 U/L showed consistent improvement, suggesting greater potential benefit among those with more pronounced tissue damage or metabolic stress. Low lymphocyte counts predicted poor response in the low-LDH subgroup, consistent with impaired immune competence.
2. **T2 – Strict Improvement**: PCT and CRP emerged as dominant factors. Extremely low PCT (<0.16 ng/mL) predicted absence of improvement—possibly reflecting individuals without systemic bacterial inflammation. Conversely, very high PCT and CRP thresholds predicted strong improvement, consistent with TPE’s effect on circulating inflammatory mediators. In particular, the branch with PCT > 0.16 ng/mL, CRP $\leq 81.26$ mg/L, and PCT $\leq 2.85$ ng/mL is classified as no strict improvement. This region corresponds to intermediate PCT without marked CRP elevation at baseline; under the strict endpoint (which requires concurrent decreases in CRP and LDH), such episodes less frequently satisfied the label in this cohort, suggesting that the strongest strict-response signal emerges primarily when baseline inflammatory burden is clearly elevated.
3. **T3 – IL-6 Early Response**: This model highlighted a clinically valuable three-zone IL-6 classification. Patients with IL-6 between 7 and 86 pg/mL—moderate inflammation—showed little benefit, a finding biologically plausible given that moderately elevated IL-6 may not be the dominant driver of pathophysiology. Conversely, patients with IL-6 > 86 pg/mL and fibrinogen ≤ 8.3 g/L formed a consistent responder group.

Combining T1, T2, and T3 allowed identification of nuanced clinical phenotypes:

“Super-responders” were consistently identified across all models and validated by Apriori rules. “Moderate IL-6, high fibrinogen” patients showed consistently poor biochemical response across models. Discordant cases (e.g., T1 = 1, T3 = 0) offered opportunities for personalized decision-making.

### Insights from Apriori rules

Association rules supplemented the trees by identifying stable, high-confidence patterns. The strongest rules converged on:

- IL-6 extremely high
- LDH high
- lymphopenia

as a constellation predicting near-certain early biochemical response within this cohort. This aligns with severe cytokine storm pathophysiology.

The Apriori rules filtered by lift metric, biomarkers and cohort characteristics are compiled in Tables 9, 10 and 11.

**Table 9:** Apriori rules (lift > 1.2)

Antecedents	Consequents	Confidence	Lift
T1, IL6_very_high, Lymph_low	T3	1.00	1.35
T1, IL6_very_high	T3	0.94	1.27
T1, LDH_high, IL6_very_high	T3	0.93	1.25
IL6_very_high, LDH_high	T1, T3	0.93	1.37
T1, LDH_high	T2, T3, IL6_very_high	0.36	1.25

**Table 10:** Biomarker thresholds derived from decision trees (data-derived split points from this cohort)

Biomarker	Threshold	Clinical Meaning
IL-6	7.24 pg/mL	Very low zone: likely improvement
IL-6	86.15 pg/mL	Very high zone: higher likelihood of IL-6 reduction when fibrinogen is low
Fibrinogen	8.31 g/L	Lower values predict IL-6 reduction
PCT	0.16 ng/mL	Extremely low $\rightarrow$ no strict improvement
PCT	2.85 ng/mL	High $\rightarrow$ predicts strict improvement
CRP	81 mg/L	High $\rightarrow$ supports strict improvement
LDH	346 U/L	High $\rightarrow$ predicts global improvement
LDH	361 U/L	Supports strict improvement
Lymphocytes	0.53 $\times 10^9$/L	Low $\rightarrow$ poor improvement
Weight	72.5 kg	Lower weight predicts improvement in Tree 1

**Table 11:** Cohort characteristics (COVID and Non-COVID)

Characteristic	COVID Cohort	Non-COVID Cohort
Number of patients	*N* ≈ 20	*N* ≈ 20
Mean age	≈60	≈55
Sex distribution	55% male	50% male
Baseline IL-6	Much higher	Moderately elevated
Baseline LDH	High	Variable
Baseline CRP	High	High
Baseline fibrinogen	High	High
TPE sessions	1–3	1–3
Improvement (T1)	$\approx 50\%$	$\approx 50\%$
Improvement (T2)	$\approx 45\%$	$\approx 45\%$
Improvement (T3)	$\approx 45\%$	$\approx 45\%$

### Comparison with ASFA Guidance

ASFA guidance provides a critical framing for how TPE should be deployed when evidence is evolving. ASFA guidelines classify apheresis indications by category and grade, distinguishing established first-line roles from contexts where the optimal role is not established and decisions should be individualized  [1]. In the COVID-19 era, ASFA operational considerations further emphasized prioritization under constrained resources and highlighted TPE-specific cautions, including the potential removal of therapeutic agents and immunoglobulins, and the importance of avoiding indiscriminate use (particularly in mild disease)  [12, 13]. Within this paradigm, TPE for severe COVID-19 is best interpreted as a selective adjunct used in patients with high-risk phenotypes—consistent with an ASFA Category III-like context where patient selection, timing, and monitoring are essential  [15].

To make this comparison actionable for protocol design, we synthesize the evidence base most frequently cited in ASFA-adjacent COVID-19 TPE discussions and map it to the operational implications of our interpretable models. Across studies, TPE is typically deployed in severe presentations (ICU-level illness), commonly targeting CRS-like or hyperinflammatory patterns and organ dysfunction, and is monitored via inflammatory and injury biomarkers (e.g., IL-6, CRP, ferritin, LDH) and oxygenation/severity indices.

ASFA operational considerations and the COVID-19 evidence base converge on the need to reserve TPE for severe phenotypes (ICU-level illness, CRS-like hyperinflammation, organ dysfunction, or specific mechanistic subgroups) rather than mild disease  [12, 15]. Published COVID-19 TPE reports repeatedly track inflammatory and injury markers (IL-6, CRP, ferritin, LDH) and oxygenation/severity measures, which aligns with our model-derived emphasis on IL-6/LDH-centered phenotypes as discriminators of biochemical response  [9, 10, 14]. Comparative evidence suggests timing may matter (earlier initiation being associated with improved outcomes in propensity-matched analysis), and many regimens use repeated exchanges rather than a single session. This supports protocol logic that treats TPE as an iterative intervention requiring reassessment after early sessions rather than a one-off rescue  [9, 11]. ASFA also notes risks relevant to TPE implementation in COVID-19, including possible removal of concurrently administered therapies and immunoglobulins. Therefore, any protocol should explicitly incorporate medication-timing coordination, careful replacement-fluid selection, and monitoring for treatment-related complications  [12, 13].

Taken together, ASFA guidance sets the governance frame (selective, individualized, resource-aware use with explicit cautions), while the COVID-19 TPE literature provides recurring practical patterns (severe phenotypes, hyperinflammatory markers, repeated exchanges, and biomarker monitoring). The most notable result from an algorithmic standpoint is that our interpretable model—derived from real ICU TPE cases—recovers essentially the same selection and monitoring axes that clinicians used in practice across these reports (e.g., IL-6/CRP/ferritin/LDH-centered hyperinflammation with organ dysfunction), supporting the use of our derived rules as a credible starting point for protocol construction and prospective audit  [9, 14, 15].

### Discussion

This study yields benefits from two major perspectives. Through our algorithmic approach, we provide an interpretable, protocol-ready decision structure that makes candidate selection and monitoring explicit. Unlike black-box prediction, the decision tree and association rules are auditable: they can be translated into concrete protocol steps such as (i) defining inclusion triggers based on severity and biomarker configurations, (ii) specifying early reassessment checkpoints after initial exchanges, and (iii) encoding stopping or escalation logic based on short-horizon biochemical response. This is particularly valuable in contexts where guidance remains conservative or individualized (ASFA Category III-like framing), because the protocol must be defensible, reproducible, and adaptable  [1, 12, 15].

From the medical perspective, the model structure clarifies a plausible “TPE-responsive” phenotype in our real ICU cohort, characterized by hyperinflammatory and tissue-injury burden markers (notably IL-6 and LDH patterns). This aligns with the recurring biomarker monitoring approach described in severe COVID-19 TPE experience, where reductions in inflammatory markers and improvements in oxygenation indices are often tracked following repeated exchanges  [9, 10, 14]. The convergence between (a) our data-driven, interpretable rules and (b) published real-world practice patterns strengthens the argument that the derived decision logic is not merely a statistical artifact; rather, it captures clinically meaningful selection heuristics that have been applied in practice.

Crucially, the modest sample size of *N* = 60 TPE episodes should be interpreted correctly. The present dataset is small, but real. That combination is appropriate for protocol seeding: producing a first version of explicit decision logic that can be implemented with oversight, prospectively audited, and iteratively refined as additional cases accumulate. In this framing, the primary deliverable is a usable decision support scaffold—transparent and reviewable—rather than a final efficacy claim. Future work should prioritize multicenter validation, standardized timing of biomarker collection relative to exchanges, and explicit accounting for confounders such as co-administered immunomodulatory agents and anticoagulation strategies, which may be affected by TPE or by the operational cautions emphasized by ASFA  [12].

## Conclusions and limitations of the Results

We present an interpretable multi-model framework that identifies biochemical phenotypes associated with near-term laboratory response following therapeutic plasma exchange (TPE) and translates those patterns into protocolizable decision logic. The work contributes on two horizons: an algorithmic approach that yields auditable rules through our three distinct decision trees that captured global, strict, and early IL-6 response profiles and distinguished responder types, and a medical perspective that clarifies a hyperinflammatory/tissue-injury phenotype consistent with severe COVID-19 TPE reports. When contextualized against ASFA guidance and COVID-era ASFA operational considerations, the resulting framework supports selective, resource-aware deployment of TPE with explicit monitoring and safety cautions. The algorithm mirrors and refines ASFA’s syndromic recommendations by introducing empirically derived biomarker thresholds, especially IL-6, LDH, fibrinogen, CRP, and PCT. Such refinement supports more precise patient selection, potentially reducing unnecessary procedures while improving outcomes in those most likely to benefit.

A central limitation of this study is the small sample size, particularly in the effective test sets used for model evaluation. In this regime, performance estimates such as accuracy and F1 score are inherently unstable: a single misclassification can change accuracy by more than $10-14$ percentage points. This is reflected in the wide 95% confidence intervals reported. While the point estimates of accuracy are consistently above 0.5, the confidence intervals are wide indicating that the available data are insufficient to estimate performance with high precision or to exclude chance-level low performance.

Due to class imbalance in several evaluation splits, a naive majority-class classifier might achieve performance comparable to the reported models. Therefore, the presented performance metrics should not be interpreted as evidence of predictive superiority, but rather as descriptive indicators of model behavior within this specific real dataset. The risk of overfitting is also substantial. Although model complexity was deliberately constrained through shallow decision trees, small-sample supervised learning remains highly sensitive to noise and sampling variability. Consequently, the specific numerical thresholds identified by the models should be interpreted as dataset-dependent approximations rather than stable or generalizable clinical decision boundaries. These thresholds are best understood as representing broader biomarker regimes rather than precise clinical cutoffs. From a purely statistical perspective, the study is underpowered for reliable estimation of generalization performance. Even resampling approaches such as cross-validation or bootstrapping would yield high-variance estimates in this setting. For this reason, we avoid claims of predictive validity and instead emphasize interpretability and clinical plausibility.

Accordingly, the present work should be interpreted as hypothesis-generating and protocol-seeding rather than as a validated predictive modeling study. Its primary contribution lies in the extraction of transparent, clinically interpretable decision logic from real-world TPE cases, providing a foundation for prospective validation.

Finally, generalizability is limited by the single-center, retrospective design and potential confounding factors, including co-administered therapies and evolving clinical practices. External validation in larger, multicenter cohorts will be necessary to determine whether the identified biomarker patterns represent reproducible response phenotypes.

Future research should validate the algorithm in multicenter cohorts, integrate outcome measures such as mortality or organ support, and explore real-time implementation in clinical informatics systems. Nevertheless, this work demonstrates that interpretable machine learning can advance evidence-based, individualized decision support for TPE as an adjunctive therapy in severe inflammatory conditions.

## Data Availability

The original clinical data used in this study are not publicly available due to the restrictions of hospital regulations and patient privacy. All data supporting the findings of this study are available upon request for noncommercial purposes from the corresponding author, typically within two weeks. The data generated in this study for the creation of figures and tables are provided with this paper. Source data are provided with this paper.
